# Dosing and switching of paliperidone ER in patients with schizophrenia: recommendations for clinical practice

**DOI:** 10.1186/1744-859X-13-10

**Published:** 2014-04-01

**Authors:** Joseph Peuskens, Gabriel Rubio, Andreas Schreiner

**Affiliations:** 1 Campus Kortenberg, University Psychiatric Centre KU Leuven, Leuvensesteenweg 517, Kortenberg 3070, Belgium; 2 Department of Psychiatry, Faculty of Medicine, Complutense University, Hospital Universitario ′12 de Octobre′, Madrid, Spain; 3 Medical & Scientific Affairs, Janssen-Cilag Europe, Middle East & Africa, Johnson & Johnson Platz 5a, Neuss 41470, Germany

**Keywords:** Schizophrenia, Antipsychotic, Paliperidone, Switching, Dosing

## Abstract

Many patients with schizophrenia receive long-term treatment with antipsychotic medication. Switching of antipsychotic medication due to lack of efficacy, tolerability issues, and partial/non-adherence is common. Despite this, consensus strategies for switching between antipsychotics are lacking. This manuscript provides practical recommendations for switching antipsychotic medication to ensure optimal management of patients with schizophrenia, with a particular focus on paliperidone extended release (ER). The authors drew on their clinical experience supported by detailed discussion of literature describing antipsychotic switching techniques and strategies and findings from paliperidone ER clinical trials. Antipsychotic switching strategies should be individualized and take into consideration the pharmacokinetic (PK) and pharmacodynamic (PD) properties of the pre- and post-switch medication. The use of temporary concomitant medications may be appropriate in some scenarios. Abrupt withdrawal of pre-switch medication may be appropriate in some instances but carries a greater risk of rebound and withdrawal symptoms than other strategies. Cross-tapering is the method most widely used in clinical practice. Paliperidone ER can be initiated without dose titration. The EU SmPC recommended dose of paliperidone ER is 6 mg/day; but doses should be individualized within the approved range of 3–12 mg/day. Higher doses may be required due to insufficient efficacy of the previous antipsychotic or in patients with acute symptoms. Recently diagnosed patients, those with renal impairment, or patients who have previously experienced tolerability issues with other antipsychotics may require lower doses. When switching from risperidone, higher doses of paliperidone ER may be required compared with risperidone. When switching from antipsychotics that have sedative and/or significant anticholinergic activity, the pre-switch antipsychotic should be tapered off gradually. Antipsychotics with less sedating and little anticholinergic activity can be tapered off over a shorter period. Temporary concomitant sedative medication may be beneficial when switching from antipsychotics with relatively higher sedative propensities. Switching from another antipsychotic to paliperidone ER requires individualized switching strategies and dosing, dependent on the characteristics of the patient and the PK and PD properties of the pre-switch medication. Cross-tapering strategies should be considered as a means of reducing the risk of rebound and withdrawal symptoms.

## Introduction

Schizophrenia is a chronic debilitating illness that negatively impacts upon virtually all aspects of patients' lives. Following a first episode, only 10%–15% of patients with schizophrenia are free from further episodes, with many patients displaying exacerbations and experiencing clinical deterioration [1]. With each relapse, remission of symptoms may be slower and the course of illness worsened [2,3]. The chronic nature of the disease means that most patients are likely to receive antipsychotic treatment for the remainder of their lives.

Discontinuation and frequent switching of antipsychotic medication are common in the treatment of patients with schizophrenia. Reasons for discontinuing or switching antipsychotic medications can include lack of efficacy [4], tolerability issues [4], partial or non-adherence to medication [2,4], relapse despite adherence to medication [5], impaired functioning [6], or patient decision [7].

The Clinical Antipsychotic Trials of Intervention Effectiveness (CATIE) [7] involved 1,492 patients randomized to receive either the oral first-generation antipsychotic (FGA) perphenazine or one of a number of oral second-generation antipsychotics (SGAs) (olanzapine, quetiapine, risperidone, or ziprasidone) for up to 18 months. This study highlighted that 74% of patients discontinued the antipsychotic medication they were assigned at randomization before the end of the 18-month period. The most common reasons for discontinuation were lack of efficacy and intolerable side effects. When compared with FGAs, SGAs are generally more effective against negative and affective symptoms with a better tolerability profile, particularly with regard to extrapyramidal symptoms (EPS) [8-13]. However, SGAs reflect a group of heterogeneous medications [14] with some considerable differences in both efficacy [15] and tolerability [16], including weight gain, metabolic changes, and prolactin-related side effects [17]. For example, a recent randomized controlled study demonstrated comparable efficacy, but at the same time, significantly reduced metabolic side effects with paliperidone extended release (ER) compared with olanzapine [18]. In addition, a survey conducted in Europe, the Middle East, and Africa suggested that 34% of psychiatrists would consider altering their patient's pharmacotherapy if they believed the patient to have impaired social functioning, while 27% of respondents indicated that switching antipsychotics would be their preferred pharmacological strategy in order to address deficits in social functioning in patients with schizophrenia [19].

In Europe, consensus guidelines suggest that the initial choice of antipsychotic medication or the decision to switch to a new antipsychotic medication should be made on the basis of individual patient preference, prior treatment response, experience of side effects, adherence history, relevant medical history and risk factors, medication side-effect profile, and long-term treatment planning [20-22]. An international consensus study [23] of antipsychotic dosing in psychiatric illness reported dosing recommendations and consensus estimates of clinically equivalent doses, including an equivalency ratio for paliperidone ER compared with chlorpromazine and with olanzapine. This study also reported a number of patient-related factors that may affect these dosing recommendations [23]. Nevertheless, consensus strategies for switching between different antipsychotic treatments are lacking. This manuscript aims to provide practical recommendations for switching antipsychotics, with a particular focus on paliperidone ER, to ensure the optimal management of patients with schizophrenia.

## Review

The authors drew on their clinical experience to develop this review manuscript, which provides practical recommendations for dosing and switching to paliperidone ER. The clinical experience is reflected within this manuscript and is supported by a detailed discussion of pertinent literature, as reviewed by the authors, including articles describing antipsychotic switching techniques and strategies, and findings from paliperidone ER clinical trials.

### Considerations when switching antipsychotic medication

Switching antipsychotic treatments can deliver potential benefits. These may include improved efficacy, tolerability, adherence, functioning, and quality of life, while reducing hospitalization rates [4].

A number of factors should be considered when switching antipsychotic treatment. Indications and contraindications to switching should be considered on an individual patient basis [5]. Other considerations include the potential risk of withdrawal symptoms [24], re-emergence of psychotic symptoms [24], perceptions of patients and their families regarding the new treatment [6], presence of stressful life events [6], and the potential introduction of new or unfamiliar adverse effects, some of which, e.g. weight gain may have a long-term negative impact on the patient's health [17,24]. It is important to carefully consider both the method of switching and follow-up plans, prior to an antipsychotic treatment switch [5]. Furthermore, it is essential to carefully review the patient's treatment history [5] and provide patient education (and educate the family/carer, if appropriate) regarding the switching procedure, potential withdrawal symptoms, and risk and signs of relapse [5,6]. Psychoeducation may also be beneficial [6]. Consideration of the pharmacokinetic (PK) and pharmacodynamic (PD) profiles of antipsychotic medications are crucial for successful titration, dosing, and switching. This includes consideration of differences in receptor binding affinities, in particular for dopaminergic, serotoninergic, alpha-adrenergic, histaminergic, and muscarinic receptors (Table 1).

**Table 1:** Approximate receptor binding profiles and half-lives of selected first-generation and second-generation antipsychotics

**Receptor**	**Paliperidone **[25]**,**[26]	**Risperidone**	**Olanzapine**	**Quetiapine**^**a**^	**Ziprasidone**	**Clozapine**	**Aripiprazole**	**Haloperidol**
Receptor binding affinity expressed as equilibrium constant (Ki)^b^								
D_2_	1.6 [25]	4 [27]	11 [27]	160 [27]	5 [27]	126 [27]	0.45 [27]	0.7 [27]
5-HT_2A_	1.1 [25]	0.5 [27]	4 [27]	295 [27]	0.4 [27]	16 [27]	3.4 [27]	45 [27]
α_1_	2.5^c^[25]	0.7 [27]	19 [27]	7 [27]	10 [27]	7 [27]	57 [27]	6 [27]
α_2_^d^	3.9^e^[25]	3 [27]	230 [27]	87 [27]	– [27]	8 [27]	– [27]	360 [27]
H_1_	19 [25]	20 [27]	7 [27]	11 [27]	47 [27]	6 [27]	61 [27]	440 [27]
M_1_	>10,000 [25]	>10,000 [27]	1.9 [27]	120 [27]	>1,000 [27]	1.9 [27]	>10,000 [27]	>1,500 [27]
Pharmacokinetic profiles, half-life								
*t*_1/2_, h	23^f^[28]	3 [29]	20–70 [30]	5–8 [30]	4–10 [30]	6–33 [30]	48–68 [30]	20 [31]
*T*_max_, h	24 [28]	1–2 [29]	5–8 [32]	1–2 [33,34]	4–6 [35]	0.4–4.2 [36]	3–5 [37]	4.9 [38]
Bioavailability, %	28 [28]	70 [29]	60–80 [30]	9 [39]	60 [30]	12–81 [30]	87 [30,37]	44–74 [31]

^a^Data refer to the quetiapine immediate release formulation; ^b^data represented as nanomolar concentration required to block 50% of receptors *in vitro* (Ki [nM]); ^c^this value relates specifically to affinity for the α_1A_ receptor; ^d^paliperidone may have higher receptor affinity for the α_2A_ receptor than risperidone [26]; ^e^this value relates specifically to affinity for the α_2A_ receptor; ^f^data refer to paliperidone extended release formulation. D_2_, dopamine type 2 receptor; 5-HT_2A_, serotonin type 2A receptor; α, alpha-adrenergic receptor; H, histaminergic receptor; M, muscarinic receptor; *t*_1/2_, half-life; *T*_max_, time at which maximum plasma drug concentration is achieved.

It is important to note that the greater the differences in receptor binding affinities between pre- and post-switch antipsychotic medications, the more care should be taken when switching. This is due to the increased risk of side effects or withdrawal symptoms or PD rebound phenomena (Table 2). For example, switching from an antipsychotic with higher affinity for cholinergic receptors (e.g., olanzapine or clozapine) or histaminergic receptors (e.g., olanzapine, quetiapine, or clozapine) to one with lower affinity for cholinergic or histaminergic receptors (e.g., risperidone, paliperidone, or aripiprazole) may cause transient rebound insomnia, anxiety, agitation, or restlessness (due to a decreased propensity for anticholinergic and/or antihistaminergic effects). A PK dopamine rebound effect may also occur when switching from a shorter half-life antipsychotic (e.g., immediate release quetiapine or ziprasidone) to a longer half-life antipsychotic, such as paliperidone ER or vice versa (Table 1).

**Table 2:** Effects of blockade of receptors and side effects that can result from withdrawal/rebound during switching

**Receptor**	**Effects of blockade**	**Potential rebound/withdrawal effect**
D_2_	Antipsychotic, antimanic, antiaggression, EPS/akathisia, tardive dyskinesia, increased prolactin	Psychosis, mania, agitation, akathisia, withdrawal dyskinesia
α_1_	Postural hypotension, dizziness, syncope	Tachycardia, hypertension
α_2_	Antidepressant, increased alertness, increased blood pressure	Hypotension
H_1_	Anxiolytic, sedation, sleep induction, weight gain, anti-EPS/akathisia	Anxiety, agitation, insomnia, restlessness, EPS/akathisia
M_1_ (central)	Negative impact on memory and cognition, dry mouth, anti-EPS/akathisia	Agitation, confusion, psychosis, anxiety, insomnia, sialorrhoea, EPS/akathisia
M_2–4_ (peripheral)	Blurred vision, constipation, urinary retention, tachycardia, hypertension	Diarrhoea, sweating, nausea, vomiting, bradycardia, hypotension, syncope
5-HT_1A_ (partial agonism)	Anxiolytic, antidepressant, anti-EPS/akathisia	Anxiety, EPS/akathisia
5-HT_2A_	Anti-EPS/akathisia, antipsychotic	EPS/akathisia, psychosis
5-HT_2C_	Increased appetite/weight	Decreased appetite

D_2_, dopamine type 2 receptor; EPS, extrapyramidal symptoms; α, alpha-adrenergic receptor; H, histaminergic receptor; M, muscarinic receptor; 5-HT_1A_, serotonin type 1A receptor; 5-HT_2A_, serotonin type 2A receptor; 5-HT_2C_, serotonin type 2C receptor. Adapted from [40].

### Switching scenarios

**Figure 1:**
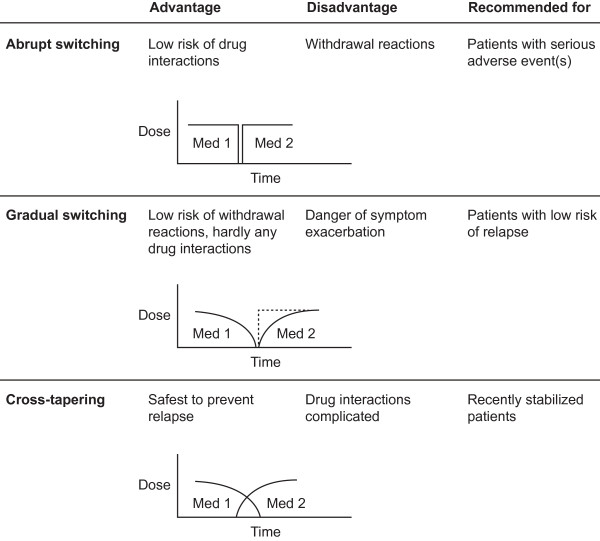
**Switching techniques for second-generation antipsychotic medication.** With kind permission from Springer Science+Business Media: CNS Drugs, Switching between second-generation antipsychotics: why and how? 19, 2005, pages 27–42. Edlinger M, Baumgartner S, Eltanaihi-Furtmuller N, Hummer M, Fleischhacker WW, Figure 1 [24].

Various methods are available for switching antipsychotic treatments [5]. These include abrupt withdrawal of pre-switch medication and initiation of post-switch medication, gradual withdrawal of pre-switch medication and initiation or titration of post-switch medication, and cross-tapering. Abrupt withdrawal of pre-switch medication and initiation of post-switch medication entails abrupt cessation of the current medication, possibly with the inclusion of a washout period, followed by immediate initiation of a new medication. The option of gradual withdrawal of pre-switch medication and initiation of post-switch medication allows for gradual tapering of the current medication, followed by the initiation of a new medication via slowly increasing doses, with or without a washout period. Finally, cross-tapering allows gradual tapering of the current medication while immediately or gradually starting a new medication [24]. These switching strategies are highlighted in Figure 1[24].

Remington et al*.*[41] conducted a meta-analysis to investigate the effect of immediate versus gradual antipsychotic switching strategies on effectiveness (as measured by Clinical Global Impression-Severity (CGI-S) and Positive and Negative Syndrome Scale (PANSS) total, positive and negative scores) and tolerability (as measured by the most common adverse event reported in each study, in addition to insomnia and somnolence). Differences in outcomes were independent of pre- and post-switch strategy. However, recent trials have shown that strategies involving a gradual reduction of pre-switch antipsychotic lead to fewer patient dropouts [42,43].

Abrupt withdrawal of pre-switch medication and initiation of post-switch medication, with or without a washout period, is the switch method most often used in randomized controlled clinical trials. In clinical trials, rescue medications, often unspecified, are frequently used in order to counter problems of abrupt switching, a practice that is less common in clinical practice [4]. Abrupt withdrawal of pre-switch medication and initiation of post-switch medication may be necessary in some cases, in particular when severe or acute adverse events with the current treatments (such as agranulocytosis, important electrocardiographic abnormalities, or other severe intolerable side effects) are observed [5]. However, abrupt withdrawal of the pre-switch medication has a greater potential for withdrawal symptoms and rebound effects [5] and carries a greater risk for relapse than more gradual withdrawal [44]. Withdrawal symptoms and rebound phenomena can be minimized by using gradual or cross-tapering approaches when the pre- and post-switch receptor affinities and/or half-lives differ considerably. However, gradual tapering may result in suboptimal dosing for a number of weeks [5]. Alternatively, in cases where the completion of the switch needs to be hastened, benzodiazepines, antihistamines, anticholinergics, gabapentin, mirtazapine, or non-benzodiazepine anxiolytics/sedatives (Table 3) can be used in a time-limited, prophylactic manner. These strategies can also minimize the risk of rebound phenomena.

**Table 3:** Managing undesirable/rebound effects at antipsychotic treatment initiation and during the initial switch period

**Side effect**	**Corrective approach or (transient) concomitant medications**
Akathisia	Lower dose, slow down switch
	Add benzodiazepine, antihistamine, beta-blocker, mirtazapine, gabapentin, low dose of low potency FGA
Mania, psychosis	Slow/reverse down titration of prior antipsychotic, increase new antipsychotic; add benzodiazepine, valproate
Agitation	Slow switch, increase dose of new antipsychotic; add benzodiazepines or low potency FGA, valproate
Anxiety	Use lower starting dose, slow switch, restrict excessive caffeine use, benzodiazepine, antihistamine, antidepressant, gabapentin
Insomnia	Slow switch, restrict excessive caffeine use, add low dose of sedating SGA, benzodiazepine, non-benzodiazepine hypnotic, antihistamine, trazodone

FGA, first-generation antipsychotic; SGA, second-generation antipsychotic. Adapted from [40].

Cross-tapering is generally the most widely used method in clinical practice but should be used with caution when the pre-switch antipsychotic has a shorter half-life (e.g., immediate release quetiapine or ziprasidone) and/or relatively greater antagonistic effects at muscarinic (e.g., quetiapine, ziprasidone, or clozapine), histaminergic (e.g., olanzapine or clozapine), or dopaminergic receptors than the post-switch antipsychotic. These considerations impact both the tapering period of the pre-switch medication (for instance, requiring prolongation) and the dosing of the new antipsychotic medication.

Switching strategies should take account of the specific medications involved; for example, switching from a sedating to a non-sedating antipsychotic requires different considerations than switching between two sedating antipsychotics. Temporary use of concomitant medications may be appropriate in some situations, for example when switching from an antipsychotic with anticholinergic or sedative properties to one without [5]. Specifically, a patient who is agitated and cannot sleep may require short-term supplementation with a benzodiazepine or another sedating medication when treatment with a non-sedating agent is initiated. This approach gives the ability to control insomnia, enabling discontinuation of the sedating medication when it is no longer needed [45]. The potential for drug–drug interactions, such as hepatic CYP-450 induction or inhibition, which occur not only when introducing but also when reducing the dose or tapering of enzyme-inducing or -inhibiting drugs, should also be considered [40].

The most appropriate switching strategy may differ for each patient and even according to the individual clinical situation within the same patient at different stages of the disease and should therefore be considered on an individual basis [21].

### Paliperidone ER: clinical efficacy

Paliperidone ER is indicated for the treatment of schizophrenia in adults as well as for the treatment of psychotic or manic symptoms of schizoaffective disorder [28,46]. The OROS^®^ technology utilized in paliperidone ER ensures stable plasma drug concentrations over a 24-h period and means that paliperidone ER may be initiated at therapeutically effective doses without the need for initial titration [47].

The efficacy and tolerability of the approved dose range of paliperidone ER (3–12 mg/day) was established in three multi-centre, placebo-controlled, double-blind, fixed-dose, 6-week pivotal trials in patients with acute symptoms of schizophrenia [48-51]. All tested doses of paliperidone ER separated from placebo from day 4 on the primary efficacy endpoint (a decrease in PANSS total scores) [28,48-50]. In all three studies, paliperidone ER was also superior to placebo on secondary endpoints; improvement in patient functioning based on the Personal and Social Performance (PSP) scale and improvement in symptoms as assessed using the CGI-S scale. In a further 6-week study, paliperidone ER (6–12 mg/day) was associated with significantly greater improvements in symptoms from baseline at 2 weeks (*p* < 0.01) and at 6 weeks (*p* < 0.05) post-initiation, in patients with acutely exacerbated schizophrenia requiring hospitalization, compared with quetiapine (600–800 mg/day) [52]. In open-label extension studies of the three key trials described above, long-term administration of paliperidone ER maintained improvements in symptoms and functioning and was generally well-tolerated for up to 52 weeks [53]. In a long-term trial designed to assess the maintenance of its effect, paliperidone ER was significantly more effective than placebo in maintaining symptom control and in extending time to relapse of schizophrenia [54]. Taken together, these studies demonstrate that paliperidone ER is an effective choice for the treatment of acute symptoms and prevention of relapse in patients with schizophrenia [8,48-50,53,54].

The studies described above used fixed dosing and washout periods in selected patient populations. The majority of fixed-dose randomized controlled studies are required for licensing purposes and provide some initial information on dosing recommendations. Two additional pragmatic studies using flexible doses of paliperidone ER in routine clinical practice were therefore conducted to complement the pivotal studies and provide further information regarding appropriate dosing in acute and non-acute patients, respectively. These studies demonstrated that flexible dosing of paliperidone ER was associated with significant improvements in symptomatology and functioning in patients with an acute exacerbation of schizophrenia [55] and in non-acute but symptomatic patients who had previously been unsuccessfully treated with oral antipsychotics [56].

### Paliperidone ER in schizoaffective disorder

In the EU, paliperidone ER is also indicated for the treatment of psychotic or manic symptoms of schizoaffective disorder in adults [28]. The efficacy and tolerability of paliperidone ER in improving psychotic and manic symptoms of schizoaffective disorder have been demonstrated in two double-blind, randomized, placebo-controlled studies [57,58]. Dosing recommendations for paliperidone ER in patients with schizoaffective disorder are 6 mg per day (some patients may benefit from higher doses, within a recommended range of 6–12 mg per day [28]) and overall, the same switching recommendations apply.

### Paliperidone ER: clinical efficacy after switching from other oral antipsychotics

The clinical benefits of switching to paliperidone ER have been demonstrated in both acute and maintenance treatment. To study these benefits in the acute setting, a 6-week flexible-dose study was performed in which 294 patients with an acute exacerbation of schizophrenia were switched to paliperidone ER (3–12 mg/day; mean initial dose, 5.8 ± 1.7 mg/day; mean mode dose, 7.7 ± 2.6 mg/day; the proportion of patients receiving 9 or 12 mg/day at study end, 33% and 21%, respectively) [55]. Results of this study demonstrated clinically relevant and statistically significant improvements from baseline in PANSS total score, patient functioning (as measured by PSP score), sleep quality, and daytime drowsiness (all *p* < 0.0001). To study the clinical benefits of switching to paliperidone ER, patients with non-acute schizophrenia, who had previously received risperidone but were sufficiently symptomatic for enrolment, were switched to paliperidone ER (3–12 mg/day). Results indicated that paliperidone ER significantly improved psychotic symptoms, disease severity, and patient functioning (as measured by PANSS, CGI-S, and PSP scores, respectively) compared with those on placebo (*p* < 0.05) [59]. In patients who were previously unsuccessfully treated with other oral antipsychotics and then switched to flexibly dosed paliperidone ER (3–12 mg/day), PANSS general psychopathological symptoms, as well as PANSS positive, negative, and total scores, significantly decreased from baseline to endpoint [56]. The main reasons for discontinuing the previous oral antipsychotic medication in these studies were lack of efficacy (56.6%), lack of tolerability (27.0%), and lack of adherence (9.1%) [56].

### Practical considerations when treating patients with paliperidone ER

#### ***Paliperidone ER receptor profile***

Paliperidone ER has a greater affinity for serotonin type 2A (5-HT_2A_) receptor relative to dopamine type 2 (D_2_) receptor and a very low affinity for muscarinic receptors [60,61]. A dose of 6–9 mg of paliperidone ER provides a mean D_2_ receptor occupancy of 70%–80%, reflecting the accepted receptor occupancy range required for optimal antipsychotic activity without increased risk of EPS [62].

#### ***Paliperidone ER recommended dose***

Three pivotal studies [48-50] of paliperidone ER examined the licensed dose range of 3–12 mg/day^a^ in patients with acute symptoms of schizophrenia. In these fixed-dose studies, the incidence of EPS was higher at doses ≥9 mg/day. Evidence from these trials suggested that a dose of 6 mg/day provides a good efficacy/tolerability ratio for a considerable number of patients [28,51]. However, clinical studies and the authors' clinical experience indicate that doses >6 mg/day may be useful for patients who are, for example acutely ill or in a post-acute phase, suffering from relevant breakthrough symptoms or who have needed higher doses of previous antipsychotic medications in the past. This is supported by a recent clinical trial in which patients suffering from an acute exacerbation of schizophrenia received flexibly dosed paliperidone ER (3–12 mg/day) for 6 weeks [54]. In addition, a number of flexible-dose studies of paliperidone ER have highlighted that, based on patients' needs, dosing is often individualized during the course of treatment [54-56]. Indeed, the flexible dosing option led to upward titration more often than downward titration [54-56,59-62]. This is also likely to reflect clinical practice.

### Paliperidone ER: switching considerations

#### ***Post-switch dosage of paliperidone ER***

Dosing of paliperidone ER following a treatment switch is likely to depend on a number of factors such as the reasons for switching antipsychotic medication, the type and dose of previous antipsychotic medication, and patient characteristics. The mean doses of paliperidone ER after switching from other antipsychotics are highlighted in Table 4.

**Table 4:** Dosing considerations when switching non-acute patients with schizophrenia from other oral atypical antipsychotics to paliperidone ER

**Prior oral antipsychotic medication**	**Daily dose of prior antipsychotic (mg)**	**Daily dose of paliperidone ER (mg)**
**Olanzapine**	10^a^	6^a^
	14.2^b^[56]	7.2^c^[63]
**Risperidone**	5^a^	6^a^
	4.3^b^[56]	7.1 [64]
**Quetiapine**	400^a^	6^a^
	485.3^b^[56]	6.8^c^[65]
**Aripiprazole**	15^a^	6^a^
	19.4^b^[56]	6.8 [66]

^a^WHO DDD (mg) [67]; ^b^last used mean daily dose; ^c^doses of up to 9 mg/day may be required depending on the individual patient circumstances [68]. WHO, World Health Organization; DDD, defined daily dose; ER, extended release.

Subgroup analyses of a large, pragmatic trial including more than 1,800 patients have shown that switching to paliperidone ER due to efficacy issues with the previous antipsychotic medication is associated with a tendency for higher paliperidone ER dosing compared with switching for other reasons, such as lack of tolerability or adherence issues [56].

In acutely ill patients, a higher baseline CGI-S score has also been demonstrated to be a significant predictor for patients requiring a higher than 6 mg paliperidone ER dose/day [68]. In addition, higher body mass index and a higher number of hospitalizations in the preceding 12 months were predictive of a mode dose of 9 mg/day in severely acutely exacerbated patients [68]. However, age, baseline PANSS total score, and baseline PSP score do not appear to be predictive of higher dosing [68].

Acutely ill patients may require higher dosages of paliperidone ER, [68] and use of paliperidone ER in the highly acute hospital setting may be associated with doses of ≥9 mg/day [55]. Furthermore, patients at imminent risk of relapse may also require higher doses [54].

Some patients may benefit from lower doses of paliperidone ER, for example patients with renal impairment [28], patients who have previously experienced side effects, or patients who show higher susceptibility to adverse effects of antipsychotics. Clinical experience of using paliperidone ER suggests that recently diagnosed patients with schizophrenia may require lower doses than those with more chronic illness [69]. Paliperidone ER is generally well-tolerated; however, akathisia and parkinsonism can occur in some patients, especially if doses in the upper range are used (≥9 mg/day) [51], although rates were lower when flexible doses of paliperidone ER were allowed and treatment could be individualized to patients' needs [56].

Paliperidone ER can be initiated at an effective dose without the need for titration [28]. However, depending on the clinical situation, post-switch titration may be useful in some patients, for example, those sensitive to or already experiencing side effects. In this case, cross-tapering of paliperidone ER and the previous antipsychotic may be useful.

#### ***Switching to paliperidone ER from oral risperidone***

In the case of switching from oral risperidone to paliperidone ER, doses that produce similar exposures of active moiety at steady state cannot necessarily be considered to be ‘therapeutically equivalent’ in the clinic [70]. A statistical analysis comparing data from several clinical trials (in the absence of a specific, direct head-to-head trial), demonstrated that higher doses of paliperidone ER are required compared to those of risperidone. This analysis suggests that 6–12 mg/day paliperidone ER produces similar efficacy to 4–6 mg/day of oral risperidone [71]. These differences in required dose may be related to the extended release profile of paliperidone following administration of paliperidone ER (peak plasma concentrations occur at 24 h) [28], considerably lower bioavailability (28% for paliperidone ER [28] versus ≥70% for oral risperidone) [29], potentially slower brain penetration due to its lower lipophilic properties [72] of paliperidone ER compared with oral risperidone. The receptor-binding profiles of risperidone and paliperidone are broadly similar, with one suggested difference being paliperidone's higher receptor affinity for the alpha 2A adrenergic (α2A) receptor [25]. Rapid withdrawal is therefore a commonly employed strategy when switching patients from oral risperidone to paliperidone ER. However, as data suggest that paliperidone ER is less sedating than risperidone [26], patients may benefit from gradual withdrawal or, if clinically indicated, temporary concomitant treatment with a sedative medication.

#### ***Switching to paliperidone ER from aripiprazole***

Aripiprazole is both a D_2_ antagonist and a partial D_2_ and 5-HT_1A_ agonist and 5-HT_2A_ antagonist, with low potential for sedation [45]. It demonstrates the highest affinity for D_2_ receptors among SGAs [25,27]. Both aripiprazole and its active metabolite dehydroaripiprazole have a long half-life, with an elimination half-life of 47 to 68 h after a single dosing of aripiprazole in healthy volunteers [73], therefore relatively rapid discontinuation of aripiprazole is possible during a treatment switch.

#### ***Switching to paliperidone ER from oral olanzapine or quetiapine***

Olanzapine and quetiapine are antipsychotics known to have sedative and anticholinergic properties. Both are potent D_2_/5-HT_2_ receptor antagonists and are also histamine H_1_ and muscarinic M_1_ receptor antagonists. H_1_ antagonism is the likely pathway for the sedating effects of these antipsychotics [74], whilst M_1_ antagonism is believed to be responsible for their anticholinergic activity. According to pharmacological principles applied in daily clinical practice and based on the PD properties of paliperidone ER (which is generally considered to have a low propensity for sedation and has a low potency at the M_1_ receptor), tapering off of these medications should be conducted over approximately 2–4 weeks (and occasionally longer), depending on the dose of olanzapine or quetiapine used and the sensitivity of the patient. As olanzapine and quetiapine have a greater propensity to cause sedation than paliperidone ER [18,52], clinical experience suggests that in individual patients, temporary concomitant use of sedative/hypnotic medications may be useful in order to avoid insomnia and anxiety when switching from oral olanzapine or quetiapine. The same principles would apply to switching from clozapine, although in clinical practice this is not expected to occur frequently.

#### ***Use of concomitant medications***

It is important that questions related to the use of concomitant medication be considered when switching antipsychotics. Paliperidone ER is not expected to cause clinically important hepatic PK interactions with drugs that are metabolized by cytochrome P450 isozymes [75]. Therefore, it is not expected to induce or inhibit clearance of drugs that are metabolized by these metabolic pathways in a clinically relevant manner.

Paliperidone ER has a low potential for sedation [18,26,51,55,59]; therefore, the use of benzodiazepines or other sedating drugs may be helpful if switching from a sedating antipsychotic to paliperidone ER. Patients who are acutely ill, agitated, and/or suffer from insomnia may need short-term supplementation with a benzodiazepine, non-benzodiazepine hypnotic, sedating SGA (e.g., quetiapine) or a low-potency FGA (e.g., levomepromazine). In a 6-week study [49], analyzing the safety and efficacy of once-daily paliperidone ER, 63–66% of patients receiving paliperidone ER received benzodiazepines as compared with 72% in the placebo group. The most commonly used benzodiazepine was lorazepam, with an average daily dose of 2.7 ± 1.2 to 3.2 ± 1.5 mg and a mean duration of 10.3–12.2 days across all treatment groups [49]. In a study of patients with an acute exacerbation of schizophrenia switching to paliperidone ER, a higher baseline CGI-S score was associated with a greater likelihood of concomitant benzodiazepine use [68]. Mean doses of benzodiazepine used were 14.4 ± 8.5 mg/day diazepam equivalents [68]. Transient concomitant use of benzodiazepine comedication for a limited period of approximately 10 days may be useful in acutely ill patients [55] and non-acute patients suffering from breakthrough symptoms or insomnia. Similarly, in the case of EPS, temporary concomitant anticholinergic medication can be used [55].

The use of concomitant medications during an antipsychotic switch should, in most cases, be temporary. It is therefore important to conduct regular clinical assessments following the switch in order to determine whether the concomitant medication is still required.

## Conclusions

Taken together, a number of studies have demonstrated that paliperidone ER is a useful option in the treatment of acute symptoms and in maintenance treatment including the prevention of relapse in patients with schizophrenia [48-53,76,77], including in those patients unsuccessfully treated with other oral antipsychotics [18,55,56,59], as well as in patients with schizoaffective disorder [57,58]. It is therefore important to consider dosing and switching strategies to ensure the optimal management of patients with schizophrenia who may benefit from a switch from another oral antipsychotic to paliperidone ER treatment. Future studies will provide additional information on switching strategies for the long-acting injectable formulation of paliperidone, paliperidone palmitate.

This manuscript outlines the recommendations for dosing paliperidone ER and for switching to paliperidone ER in patients with schizophrenia in different clinical situations. Data from recent flexible dose studies in real-world clinical settings have demonstrated that patients will have differing dose requirements based on individual needs [55,56,59], with an appropriate switching strategy for patients who have previously been unsuccessfully treated with other oral antipsychotics. Reasons for switching can include lack of efficacy, poor tolerability, partial or non-adherence to medication, or patient wish. The pharmacological profile of paliperidone ER, the type and dose of the previous antipsychotic(s), the clinical situation (e.g., acute/non-acute) and the individual patient profile (e.g., severity of symptoms, sensitivity to side effects, previous treatment responses, or need for higher dosages) should be taken into account when switching patients with schizophrenia or schizoaffective disorder.

## Endnotes

^a^A higher dose of 15 mg/day was also included in one of the clinical trials [48]; however, this dose is outside of the approved dose range.

## Competing interests

JP has received speaker's honoraria and research support from and was part of advisory boards for AstraZeneca, Bristol-Myers Squibb, Eli Lilly, Janssen-Cilag, and Lundbeck. AS is a full-time employee of Janssen-Cilag Medical and Scientific Affairs Europe, Middle East and Africa, and is a shareholder of Johnson & Johnson. GR is teacher of Psychiatry at Complutense University and has been MD consulting for Bristol-Myers, Eli Lilly, Janssen-Cilag, Lundbeck, and Pfizer.

## Authors’ contributions

All authors (JP, AS, and GR) contributed to the conception, design, and development of the manuscript. All authors read and approved the final manuscript.
